# Genomic distribution of SINEs in *Entamoeba histolytica* strains: implication for genotyping

**DOI:** 10.1186/1471-2164-14-432

**Published:** 2013-07-01

**Authors:** Vandana Kumari, Lakshmi Rani Iyer, Riti Roy, Varsha Bhargava, Suchita Panda, Jaishree Paul, Jaco J Verweij, C Graham Clark, Alok Bhattacharya, Sudha Bhattacharya

**Affiliations:** 1 School of Environmental Sciences, Jawaharlal Nehru University, New Delhi 110067, India; 2 School of Life Sciences, Jawaharlal Nehru University, New Delhi 110067, India; 3 School of Computational and Integrative Sciences, Jawaharlal Nehru University, New Delhi, India; 4 Laboratory for Medical Microbiology and Immunology, Laboratory for Clinical Pathology, St. Elisabeth Hospital, Tilburg, The Netherlands; 5 Department of Pathogen Molecular Biology, London School of Hygiene and Tropical Medicine, Keppel Street, London, WC1E 7HT, UK

**Keywords:** *Entamoeba histolytica*, Genotype, EhSINE1, SINE occupancy, Polymorphism, Strain typing

## Abstract

**Background:**

The major clinical manifestations of *Entamoeba histolytica* infection include amebic colitis and liver abscess. However the majority of infections remain asymptomatic. Earlier reports have shown that some *E. histolytica* isolates are more virulent than others, suggesting that virulence may be linked to genotype. Here we have looked at the genomic distribution of the retrotransposable short interspersed nuclear elements EhSINE1 and EhSINE2. Due to their mobile nature, some EhSINE copies may occupy different genomic locations among isolates of *E. histolytica* possibly affecting adjacent gene expression; this variability in location can be exploited to differentiate strains.

**Results:**

We have looked for EhSINE1- and EhSINE2-occupied loci in the genome sequence of *Entamoeba histolytica* HM-1:IMSS and searched for homologous loci in other strains to determine the insertion status of these elements. A total of 393 EhSINE1 and 119 EhSINE2 loci were analyzed in the available sequenced strains (Rahman, DS4-868, HM1:CA, KU48, KU50, KU27 and MS96-3382. Seventeen loci (13 EhSINE1 and 4 EhSINE2) were identified where a EhSINE1/EhSINE2 sequence was missing from the corresponding locus of other strains. Most of these loci were unoccupied in more than one strain. Some of the loci were analyzed experimentally for SINE occupancy using DNA from strain Rahman. These data helped to correctly assemble the nucleotide sequence at three loci in Rahman. SINE occupancy was also checked at these three loci in 7 other axenically cultivated *E. histolytica* strains and 16 clinical isolates. Each locus gave a single, specific amplicon with the primer sets used, making this a suitable method for strain typing. Based on presence/absence of SINE and amplification with locus-specific primers, the 23 strains could be divided into eleven genotypes. The results obtained by our method correlated with the data from other typing methods. We also report a bioinformatic analysis of EhSINE2 copies.

**Conclusions:**

Our results reveal several loci with extensive polymorphism of SINE occupancy among different strains of *E. histolytica* and prove the principle that the genomic distribution of SINEs is a valid method for typing of *E. histolytica* strains.

## Background

*Entamoeba histolytica,* the etiological agent of amoebiasis, is a protistan parasite that lives in the human intestine. Amoebiasis is the third leading cause of death due to parasitic disease [1]. According to the WHO, about 40–50 million people are infected annually causing approximately 100,000 deaths worldwide. About 90% of the infections with this parasite remain asymptomatic [2].What leads to the varied outcome of infection is not known, but it is possible that the genotype of the strain influences the outcome [3]. The suggestion has been made that inherently avirulent strains exist that may be associated with unique genotypes [4]. The *E. histolytica* strain Rahman is considered to be avirulent in axenic culture since it shows reduced cytopathic activity on epithelial cells and does not form liver abscesses in animal models [5,6]. Data are, however, insufficient to assign virulence properties to specific genotypes of *E. histolytica.*

Retrotransposons without long terminal repeats are generally called long interspersed nuclear elements (LINEs) and their short non autonomous partners are called SINEs [7]. LINEs are generally ~5 kb in length and encode the functions required for retrotransposition, while SINEs are short and do not code for proteins. They utilize the LINE-encoded proteins for their own retrotransposition. Both LINEs and SINEs are efficient genome invaders and are widespread in eukaryotes [8]. In *E. histolytica* the EhLINEs (4.8 kb) and EhSINEs (0.5 to 0.7 kb) constitute 11.2% of the genome [9]. They belong to three closely related families, of which EhLINE1/EhSINE1 are the most abundant. These elements are present mostly in the intergenic regions [10,11], with a T- rich sequence within 50 bp upstream of the site of insertion [10,12]. Due to their mobile nature they can occupy different genomic locations and may influence the phenotype of the organism by activating or silencing the genes in their vicinity. Previous work has shown that a number of SINE1 occupied sites in *E. histolytica* are unoccupied in the non pathogenic species *Entamoeba dispar* and *vice versa *[11,13,14] which may have important consequences for the pathogenicity of the parasite.

A number of studies in different organisms have utilized SINEs as useful markers for phylogeny [15]. It has been argued that SINE insertion analysis is one of the best methods for determining relationships of closely related species since SINEs are widely dispersed in the genome and, unlike DNA transposons, there is no evidence of any process that removes SINEs from the genome once they are inserted. Nonspecific SINE deletions due to unequal crossing over are relatively rare. Thus the absence of a SINE at a particular locus signifies the ancestral state. The probability of independent insertions at the same locus is exceedingly low, which links SINE-containing loci as related by descent [16,17]. For these reasons population genetic analysis can be performed more accurately with SINEs than with RFLPs and microsatellite loci (where the same allele may be shared by two individuals by chance). Here we have explored the possibility of using EhSINE insertions as strain-specific markers.

Several methods have been developed for the genotyping of this parasite [18-24], which have their individual limitations. Polymorphisms are observed in short tandem repeat numbers, and repeat sequences present in the genes encoding chitinase and the surface antigen SREHP, as well as in the arrays of tRNA genes of *E. histolytica*. These have been utilized successfully for strain identification [25,26]. However the size variation in most of these loci is small, sometimes making it difficult to detect polymorphism by agarose gel electrophoresis, so DNA sequencing is normally used for confirmation. A transposon display technique was also devised for strain identification based on the genomic distribution of EhSINE1 [27]. However, this method is not suitable for use with clinical isolates.

Here we analysed 393 EhSINE1 and 119 EhSINE2 loci present in the HM-1:IMSS strain of *E. histolytica* for insertion polymorphism in other sequenced strains (http://www.Amoebadb.org) [28,29]. Seventeen loci were found (13 for EhSINE1 and 4 for EhSINE2) that showed insertion polymorphism. Of these, six loci were validated experimentally in strain Rahman. Three of these loci were tested in 7 other axenically grown strains and 16 clinical isolates. Each of the loci gave a single specific amplicon with the primer sets used, making this a suitable method for genotyping. We also report a bioinformatic analysis of EhSINE2 elements.

## Methods

### Analysis of polymorphic loci

The *E. histolytica* HM-1:IMSS genome sequence is available in 1529 scaffolds as the full genome could not be assembled into chromosomes. The sequences were downloaded from NCBI [accession number AAFB00000000]. Different strains of *E. histolytica,* namely HM1:CA, DS4-868*,* KU27*,* KU48*,* KU50*,* MS96-3382 and Rahman were downloaded from AmoebaDB (http://www.amoebadb.org) [29]. These are partially assembled sequences obtained using next generation sequencing technologies.

Table 1 shows statistics of the genome sequences used in the study*.* A database of EhSINE1 elements was built based on the results generated by Huntley *et al.*[30]. A total of 393 EhSINE1 elements were included. Elements that were less than 450 bp were omitted. Flanking sequences of 1000 bp from both 5′- and 3′-ends of all EhSINE1 elements were extracted using a perl code. The flanking sequences were mapped separately to the contigs of the various strains of *E. histolytica* using BLAST [31] and only when both flanking sequences of a specific SINE element mapped to a single contig was it used for further analysis. Presence of EhSINE1 was scored when the distance between the flanking sequences in the target strain was found to be greater than or equal to 450 bp. On the other hand, if the distance between the flanking pairs was less than or equal to 100 bp then the SINE was considered to be missing. All results were validated by manual inspection. Similarly all the EhSINE2 copies having a length greater than 400 bp and similarity of more than 70% with the EhSINE2 consensus sequence [32] were extracted from the *E. histolytica* HM-1:IMSS genome. This resulted in 119 EhSINE2 copies, which were analysed for their locus occupancy in the various sequenced strains.

**Table 1:** **Genome sequence data of different strains of *E. histolytica *used in this study**

**Strains**	**Bases**	**Number of scaffolds**
*Entamoeba histolytica* HM-1:IMSS (REFERENCE)	20835395	1529
*Entamoeba histolytica* DS4-868	19757076	1180
*Entamoeba histolytica* KU27	19648908	1178
*Entamoeba histolytica* KU48	16681302	1172
*Entamoeba histolytica* KU50	11894619	1100
*Entamoeba histolytica* MS96-3382	19016113	1171
*Entamoeba histolytica* Rahman	17583380	1145
*Entamoeba histolytica* HM1:CA	17729886	1172

**Axenic and xenic cultivation of *E. histolytica***- Axenic strains HM-1:IMSS and Rahman were maintained by continuous subculturing in TYI-S-33 medium [33], and the rest of the axenic strains were maintained in LYI-S-2 medium [34]. Xenic strains were maintained by continuous subculturing in Robinson′s medium [35].

**Genomic DNA isolation**- Genomic DNA of axenic and xenic *E. histolytica* strains was isolated using a genomic DNA isolation kit (Promega, USA) and the QIAamp® DNA Mini Kit (Qiagen, Germany), respectively, according to the manufacturer’s instructions.

**Polymerase chain reaction (PCR) -** Primers were designed from the flanking sequences of different EhSINE1 copies obtained from the *E. histolytica* HM-1:IMSS database (Additional file 1: Figure S1). All PCR reactions were performed with Biotools DNA polymerase (Biotools, B&M Labs, Spain); the PCR programme consisted of initial denaturation for 5 min at 94°C followed by 30 cycles of 30 sec at 94°C, annealing for 30 sec at a temperature dependent on the T_m_ of the primers used, and an extension time at 72°C dependent on the size of amplicon. Products were resolved on a 1% agarose gel (USB, Spain) containing 0.5 μg/ ml of ethidium bromide using 0.5X TBE (Tris borate EDTA pH8) buffer.

**Southern blotting and hybridization**- DNA was transferred to HYbond™-N + Nylon membrane (GE Healthcare) using standard methods [36]. Labeled probes were prepared using α-^32^P–dATP by the random priming method using the NEBlot^(R)^ kit (NEB, USA) according to the manufacturer’s instructions. Blots were hybridized overnight with probe at 65°C in a solution of 1% SDS, 1 M NaCl and 100 μg/ml of salmon sperm DNA, washed to remove nonspecific probe, exposed (Fujifilm) and scanned by phosphorimager.

**DNA sequencing**- Amplicons were extracted from agarose gels using a gel extraction kit (Qiagen) and cloned into the pGEM-T vector (Promega, USA). Sequences were generated commercially (TCGA, India) and compared using ClustalW software (Bioedit).

**Analysis of Target Site duplication (TSD) and internal repeats (IRs) using MEME-** The online tool MEME [37] was used for the analysis of TSDs and IRs of SINE2. 50 bp of sequence upstream and downstream of the EhSINE2 were extracted from the *E. histolytica* HM-1:IMSS genome and these were analysed for TSD. Since the longest TSDs found were in the range of 16–20 bp, and some of the shorter TSDs may result from accumulation of mutations in older SINE insertions, TSDs having size < 8 bp were excluded. The input consisted of 79 FASTA formatted sequences of TSDs with the default settings of width (Minimum 6 and Maximum 50) and the search was optimized for identifying zero or one motif per sequence. For IR analysis 150 sequences were subjected to MEME analysis in a similar way.

## Results and discussion

### Identification of genomic loci with differential EhSINE1/EhSINE2 occupancy in the sequenced *E. histolytica* strains

The availability of genome sequences of a number of *E. histolytica* strains is likely to help define the level of polymorphism in SINE distribution in *E. histolytica*. EhSINE1 (445 copies) and EhSINE2 (256 copies) constitute the majority of the SINE population of *E. histolytica*. There are only 49 copies of EhSINE3 [9], therefore we focused only on EhSINE1 and EhSINE2 for this study. Out of 445 copies of EhSINE1, 393 are full-length (>450 bp) [31], and only full length copies were used for analysis. We performed a similar analysis with EhSINE2 and found 119 full length copies (length >400 bp and similarity >70% with the EhSINE2 consensus) in strain HM-1:IMSS.

**Figure 1:**
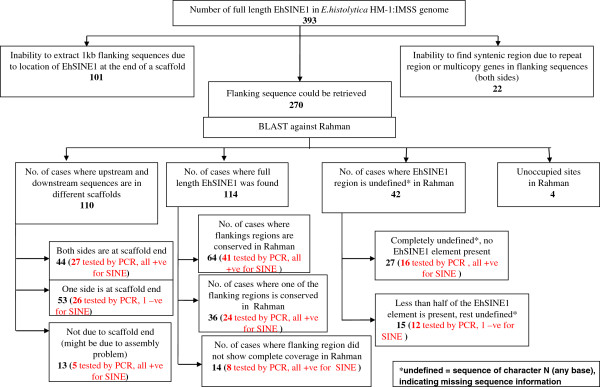
**Analysis of EhSINE1: 393 EhSINE1 copies (length > 450 bp) of *Entamoeba histolytica *HM-1:IMSS were taken for analysis.** 1.0 kb from both 5′- and 3′-ends of each EhSINE1 element were extracted using a perl code wherever possible. The flanking sequences were separately mapped to the contigs of the Rahman strain using BLAST. Only when both flanking sequences of a specific SINE element mapped to a single contig was it used for further analysis.

**Figure 2:**
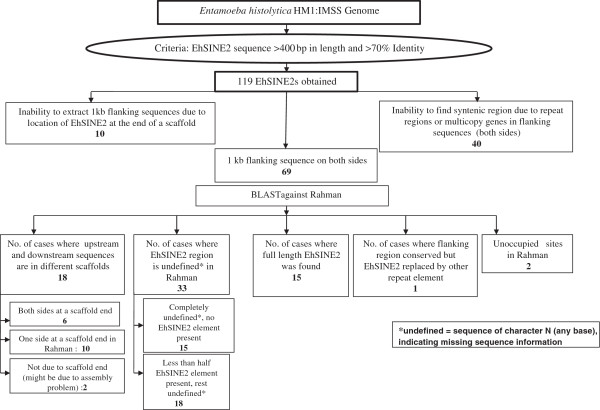
**Analysis of EhSINE2.** All the copies of EhSINE2 fulfilling the above mentioned criteria, were extracted from the whole genome of *Entamoeba histolytica* HM-1:IMSS. These were compared with the Rahman database using BLAST. Loci identified were analyzed for their occupancy as described.

Insertion polymorphism of EhSINEs 1 and 2 was detected by comparing the genomic location of all full length copies in strain HM-1:IMSS with the same loci in strain Rahman (which has lost virulence in axenic culture). Flanking sequences surrounding each SINE (1 kb from both sides) were taken into consideration in identifying the SINE-containing loci. An element was considered to be present when along with SINE the flanking sequences were the same in the two strains. The results of this analysis are presented in Figures 1 and 2. Out of 393 full length EhSINE1 copies it was possible to do this analysis for only 270 due to an inability to extract one of the flanking sequences for the rest, because either the SINE was present at the end of the scaffold or was flanked by repetitive sequences (Figure 1). Further, out of these 270 copies, full length EhSINE1 copies could be clearly mapped in Rahman in only 114 cases; in others this was not possible as the upstream and downstream sequences were in different scaffolds of Rahman. Additionally, we did not consider 42 EhSINE1 loci as there were undefined nucleotides at many positions. Finally, we found 4 loci where the flanking sequences in strains HM-1:IMSS and Rahman were conserved but the EhSINE1 sequences were completely missing in Rahman, as against 114 loci where EhSINE1 was present in both strains.

Similarly, out of the 119 full-length copies of EhSINE2 it was possible to use only 69 copies for our analysis (Figure 2), and only 2 unoccupied sites were identified in Rahman following the criteria described for EhSINE1. Since the total number of unoccupied sites obtained was rather small (4 out of 270 for EhSINE1, and 2 out of 119 for EhSINE2), we checked to see if we were missing some polymorphic loci in the copies that could not be computationally analyzed. PCR primers were designed using the genes flanking a number of EhSINE1 loci in HM-1:IMSS and were used to amplify the same loci from genomic DNA of strain Rahman. A total of 159 loci were tested from the various categories listed in Figure 1. Of these, the amplicon size in Rahman was identical with HM-1:IMSS at 157 loci, showing that these loci were all occupied, while at the remaining two loci (17 and 19) the EhSINE1 was absent from Rahman. Locus 17 was missed in the computational analysis because the sequence of the SINE, and some sequence upstream of it, contained undefined nucleotides in Rahman. In the case of locus 19 the corresponding sequence was located in three different contigs in Rahman. Therefore the combined experimental and computational analysis allowed us to identify 6 EhSINE1 loci that are polymorphic between strains HM-1:IMSS and Rahman.

A number of *E. histolytica* strains (DS4-868, KU27, KU48, KU50, MS96-3382, HM1:CA), for which Next Generation Sequencing (NGS) data are currently available, were analyzed using the approach described above. Since NGS output is in the form of short sequence reads which are assembled into a large number of scaffolds, it is likely that a number of polymorphic sites were missed in this analysis. A total of 17 polymorphic loci (13 EhSINE1 loci and 4 EhSINE2 loci) were found (Table 2). Out of the 17, 9 loci were polymorphic in more than one strain. The results suggest that SINE insertion polymorphism is widespread among strains and isolates of *E. histolytica*. Analysis of sequence in the database at sites where the SINEs were scored absent showed that in some cases a small fragment of the SINE sequence was still present, and in some others a part of the flanking sequence was missing (Table 2). We cross-checked this by sequencing some of these loci in Rahman and present evidence below that there was actually no SINE sequence left at these loci, and the reported sequence in the database was erroneous. Such assembly errors may be expected when dealing with highly repetitive sequences. We have not cross-checked all the loci and cannot comment on the status of these.

**Table 2:** SINE polymorphic loci in sequenced strains (AmoebaDB)

**Strain**	**Scaffold ID in HM1:IMSS**	**Position of Sine in HM1**	**Scaffold ID in strain**	**Comments**
**SINE1**				
**Rahman**	DS571157 (locus 13) ★	85456-86055	EhRm_Scaffold01127	
	DS571247 (locus 17) ●	15166-14637	EhRm_scaffold00561	90 bp of SINE present *
	DS571226 (locus 19) ▲	27013-26485	EhRm_scaffold00536	
			EhRm_contig21711	
	DS571158 (locus 42)	27165-27700	EhRm_scaffold00892	
	DS571410 ⬌	9760-9214	EhRm_scaffold01072	
	DS571210	20547-21121	EhRm_scaffold00002	
**DS4**	DS571410 ⬌	9760-9214	EHDS4_2898	
	DS571358 ➞	20913-21457	EHDS4_2995	
**KU27**	DS571358 ➞	20913-21457	EHKU27_2995	
**KU48**	DS571410 ⬌	9760-9214	EHKU48_2898	
	DS571979	698-1191	EHKU48_3914	
	DS571226 ▲	27013-26485	EHKU50_3346	40 bp of SINE present
**KU50**	DS571145 ⧗	332477-331932	EHKU50_3878	
	DS571175	74276-74805	EHKU50_4078	
**MS96**	DS571366	21421-20852	EHMS96_3899	50 bp of SINE from 5’ end present
	DS571247 ●	15166-14637	EHMS96_2840	Truncated from both side (397 bp present) *
	DS571145 ⧗	332477-331932	EHMS96_3878	150 bp upstream flank also missing
	DS571358 ➞	20913-21457	EHMS96_2995	60 bp downstream flank also missing
	DS571157 ★	85453-86052	EHMS96_4213	Only 80 bp of SINE present*
**HM1:CA**	DS571426	1546-2044	EHHM1_CA_4170	312 bp upstream and 275 bp downstream flank missing
	DS571487	12336-12883	EHHM1_CA_3091	60 bp of SINE from 5’ end present and 500 bp downstream flank missing
**SINE 2**				
**Rahman**	DS571418 (locus 18) ↷	2292-1614	EhRm_scaffold00754	
	DS571150 (locus 50) ♦	145690-146349	EhRm_scaffold00159	
**DS4**	DS571569	871-1494	EHDS4_3206	
**KU48**	DS571418 ↷	2292-1614	EHKU48_4206	
**KU50**	DS571418 ↷	2292-1614	E EHKU50_4206	
	DS571145	365212-365664	EHKU50_3878	
**MS96**	DS571569	871-1494	EHMS96_3206	
	DS571150 ♦	145690-146349	EHMS96_3252	

13 EhSINE1 and 4 EhSINE2 loci of HM-1:IMSS were unoccupied in the various sequenced strains (Rahman, DS4-868, KU27, KU48, KU50, MS96-3382 and HM1:CA). Identical unoccupied loci in different strains have been indicated by identical shapes.

*These loci have been tested in the respective strains and found completely unoccupied, in conflict with the sequence data available in AmoebaDB.

**Figure 3:**
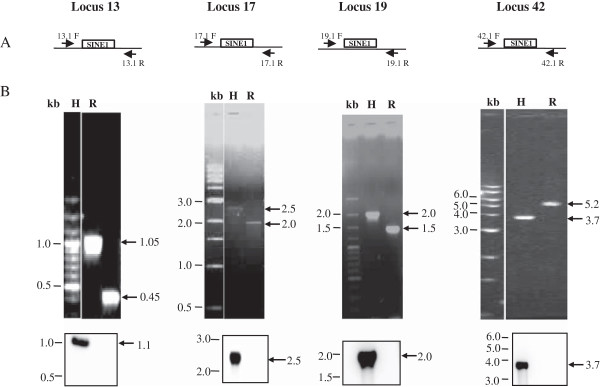
**Detection and validation of EhSINE1 polymorphic loci 13, 17, 19 and 42. (A)** Schematic representation of primers designed from different loci. The hollow box represents the EhSINE1 element; flanking genes have not been shown for simplicity. **(B)** PCR was performed using genomic DNA of HM-1:IMSS (H) and Rahman (R) strains as template, using primers from sequences flanking the EhSINE1 copy as shown in the schematic representation. The size of amplicons was determined by electrophoresis in 1% agarose gels (Top panel). Of 4 SINE1 unoccupied sites found computationally two were tested (13 and 42). Two more (17 and 19) were evaluated by PCR and Southern Blotting in Rahman. The sizes of amplicons obtained are indicated on the right, with arrows. The amplicon from strain Rahman was shorter by ~550 bp (the size of EhSINE1) at loci 13, 17 and 19, but was longer at locus 42 (explained in the text). The absence of EhSINE1 was further confirmed by Southern blotting with EhSINE1 probe, which failed to hybridize with the amplicons from strain Rahman (Bottom panel).

**Figure 4:**
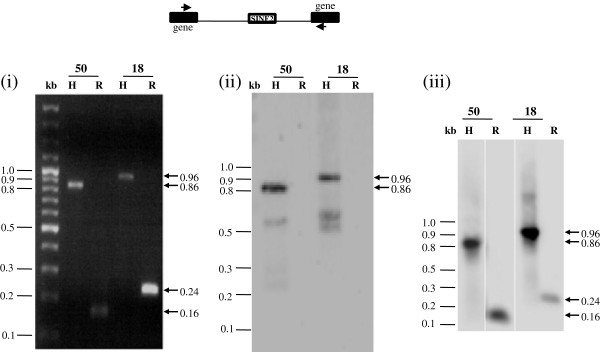
**Validation of EhSINE 2 polymorphic loci 18 and 50.** PCR was performed using genomic DNA of *E. histolytica* HM-1:IMSS (H) and Rahman (R) as template, using primers from sequences flanking the EhSINE2 copy (number as shown on top). The size of amplicons was determined by electrophoresis in 1% agarose gel. EhSINE2 was missing in Rahman at the two loci, as the amplicon from Rahman was shorter by ~700 bp (the size of EhSINE2) at these loci (Panel **(i)**). The sizes of amplicons obtained are indicated on the right (arrows). The absence of EhSINE2 was further confirmed by Southern blotting with EhSINE2 probe, which failed to hybridize with the amplicons of strain Rahman (Panel **(ii)**). The specificity of the amplicon in Rahman was checked by Southern blotting with locus specific probe (Panel **(iii)**), which hybridized with the amplicons in both strains.

Of the eight predicted polymorphic loci in strain Rahman we validated experimentally six using PCR (Figures 3 and 4) with primers designed from the flanking sequences of EhSINE1/EhSINE2 in HM-1:IMSS (Additional file 1: Figure S1 and Additional file 2: Table S1)*.* The absence of SINE sequences was inferred from the size of the amplicon (smaller by the size of SINE) and by Southern hybridization using a SINE sequence as a probe. The amplicon sizes in Rahman from three EhSINE1 polymorphic loci (13, 17 and 19) were smaller by about 550 bp suggesting that indeed these sites lacked EhSINE1. This was also confirmed by Southern hybridization (Figure 3B, bottom panel). In contrast, the amplicon size of another polymorphic EhSINE1 locus (42) was actually larger by 1.5 kb in Rahman. Probing a Southern blot of the amplicon using EhSINE1-flanking sequences from locus 42 confirmed that the amplified region in Rahman indeed belonged to the same locus (Additional file 3: Figure S2). However, two different sets of primers designed using the HM-1:IMSS sequence at this locus failed to produce an amplicon in Rahman. Therefore it appears that this locus may have undergone multiple changes and is not a simple case of SINE absence. We did not analyse this locus further. The two predicted EhSINE2 polymorphic loci (18 and 50) were also validated using PCR and Southern hybridization (Figure 4 ii and iii). At both loci the amplicons from Rahman were 700 bp shorter (the size of EhSINE2).

These loci were also found to be polymorphic among different strains and isolates of *E. histolytica* as deduced from analysis of NGS data (compiled in Additional file 4: Table S2). In some strains, although the SINE was present at the locus, the sequence showed some truncations or short deletions. If these changes are not due to assembly errors in the database one could envision various factors that may contribute to this. Most of the truncations were at the 5′-end of the SINE, which could result from the well known phenomenon of incomplete reverse transcription of the SINE RNA template during retrotransposition [38]. Short deletions may appear due to recombination between genomic SINE copies, or due to replication slippage at the short internal repeats in the EhSINEs (described later). However, some of these changes are, indeed, due to sequence assembly errors in the database, which we document below for locus 17 in strains Rahman and MS96-3382.

### Sequence analysis of some of the polymorphic loci in strains HM-1:IMSS and Rahman

Sequence data available for the two genomes in AmoebaDB shows that the assembled genome data of Rahman has many more undefined regions and gaps. There are 1529 scaffolds defining the HM-1:IMSS genome (in the size range of 0.9 kb-500 kb) compared to 1145 of Rahman (in the size range of 2 kb-170 kb) and 17378 unassembled contigs. We examined the sequences at loci 13, 17, 19 and 42 more closely and found that the locus 13 sequence was located in a single scaffold in both strains and the sequence was identical except for the loss of EhSINE1 in Rahman. However, the sequences at the other loci were either found in multiple scaffolds/contigs in Rahman, or contained undefined regions, as described below.

**Figure 5:**
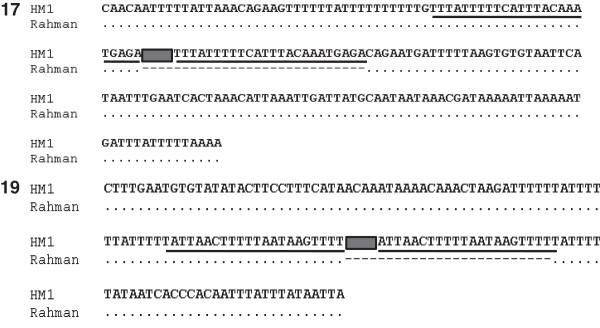
**Sequence alignment of EhSINE1 Loci 17 and 19.** Genomic DNA of HM-1:IMSS and Rahman was used to obtain amplicons of the two loci, which were cloned and sequenced. Underlined sequences correspond to the target site (site of EhSINE1 insertion), which is duplicated in HM-1:IMSS and present as single copy in Rahman. SINE1 has been represented by solid box, dotted line shows conserved sequence and broken line represent missing sequence of Rahman with respect to HM-1:IMSS.

Locus 17 was present in scaffold DS571247 (HM-1:IMSS) and EhRmscaffold_00561 (Rahman). Closer examination showed that although most of the EhSINE1 sequence was missing at this locus in Rahman, a stretch of 84 bp still remained at the 5′ end (Additional file 4: Table S2 and Additional file 5: Figure S4). This was followed by a large region of undefined sequence (~750 bp), and if this is an accurate estimate of its size we should obtain amplicons of similar size in both strains. However our data clearly showed that the amplicon in strain Rahman was shorter by 0.5 kb and it did not hybridize with a probe from EhSINE1 sequence (Figure 3). To further verify our results we cloned and sequenced these amplicons from both the strains. Sequence comparison showed that the entire stretch of EhSINE1 was missing in Rahman (Figure 5). EhSINE1 insertion is typically accompanied by target site duplication (TSD) and the Rahman sequence had only one copy of the TSD seen in HM-1:IMSS. The rest of the flanking sequence was identical in the two strains. The 84 bp piece of EhSINE1 shown in the database at this locus was not found in our sequence; rather the entire EhSINE1 was missing. We believe this discrepancy could have arisen due to assembly errors in the database.

Locus 19 was present in the scaffolds DS571126 (HM-1:IMSS) and EhRmscaffold_00536 (Rahman). The sequence upstream of the EhSINE1 location in HM-1:IMSS was undefined in Rahman. However we found three unassembled contigs (EhRmcontig_00303, EhRmcontig_00523 and EhRm_contig21711) in the Rahman database that matched the HM-1:IMSS sequence (Additional file 6: Figure S5). An amplicon from Rahman generated by PCR amplification using a primer each designed from EhRmcontig_00523 and EhRcontig_21011 displayed the expected size (Figure 3B), showing that these contigs likely belong to this locus. Sequence analysis of the amplicon confirmed that the two strains were identical except for the loss of EhSINE1 in Rahman (Figure 5).

Locus 42 in HM-1:IMSS was in one scaffold (DS571158), while in Rahman the syntenic sequence was present across three different scaffolds/contigs (Additional file 7: Figure S3). One contig spanned the downstream gene sequence with which primer 42.1 R was an exact match. However, in primer 42.1 F (Additional file 7: Figure S3) the 3′ nucleotide was a mismatch. Sequence comparison of this region revealed single nucleotide differences at several positions, which may explain our failure to amplify this locus from Rahman using HM-1:IMSS primers.

These results suggest that some of the sequence data currently available in the database needs reanalysis and the predictions need to be validated by experimentation. Our analysis has helped to correctly assemble the sequences at loci 17, 19 and 42 in Rahman.

### Genotyping using SINE sequences

**Figure 6:**
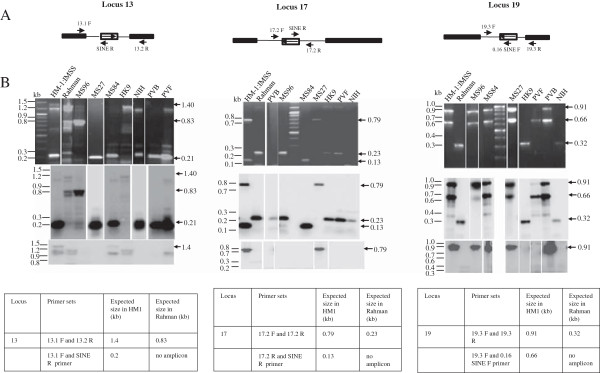
**Categorization of strains based on EhSINE1 loci 13, 17 and 19: (A).** Schematic representation of primer positions in each locus. Solid boxes represent the flanking genes, hollow box represents EhSINE1 element and the arrow inside it shows the orientation of EhSINE1 with respect to the locus. **(B)** PCR was performed using the two primer pairs indicated in the Tables below, with the genomic DNA of different strains of *E. histolytica* as template. For each locus and strain PCR reactions using the two primer sets were mixed and resolved on a 1% agarose gel (upper panel); the gel was subjected to Southern blotting and hybridized with the locus-specific probe to check the specificity of the band pattern (middle panel). Hybridization was then performed with the EhSINE1 probe to check for the presence or absence of EhSINE1 in these loci in different strains (lower panel). The expected size of the amplicons for each locus is given in the tables at the bottom of the figure.

**Figure 7:**
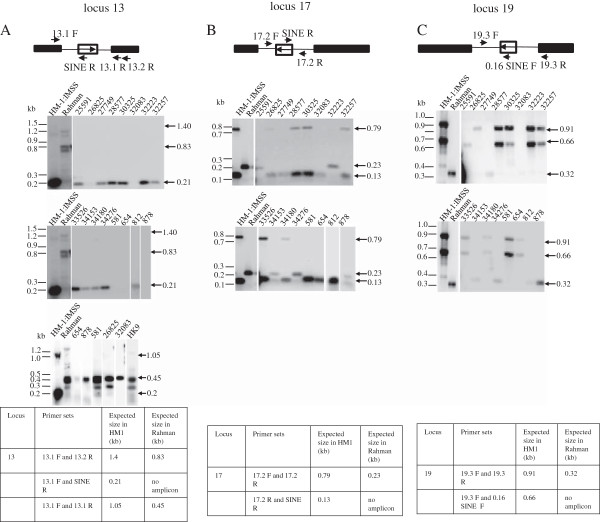
**Strain identification in xenic cultures based on locus 13, 17 and 19: PCR was performed using genomic DNA of 16 different xenic cultures of *E. histolytica *for each locus, as described in Figure** 6**.** PCR reactions were resolved on a 1 % agarose gel and subjected to Southern blotting with the locus specific probes 13 (Panel **A**), 17 (Panel **B**), or 19 (Panel **C**). Samples which did not give a product at locus 13 were amplified using an alternate reverse primer 13.1 R instead of 13.2 R followed by Southern blotting and hybridization with a locus specific probe. The expected size of the amplicon with each primer set is mentioned in the table below each locus panel.

We explored the possibility of using some of the polymorphic loci as markers for genotyping. For this we focused on loci 13, 17 and 19 and tested them using 23 axenic and xenic strains of *E. histolytica*. A genotyping method would need to be used for patient samples, where large amplicons may be difficult to obtain reproducibly due to impurities in DNA preparation and low *E. histolytica* DNA concentrations. We therefore designed primers as close to the EhSINE1 insertion site as possible to minimize amplicon size (Additional file 1: Figure S1). For each locus two primer sets were used; one set was designed from flanking sequences and the other set comprised one of the flanking primers combined with a primer from the EhSINE1 sequence (Figure 6A and Additional file 2: Table S1). Although care was taken to design primers for each locus that did not match the *Entamoeba dispar* genome, this was not possible in all cases due to extensive sequence conservation between the two species. However one primer from each pair for all three loci had no match in *E. dispar* (Additional file 2: Table S1). The amplicons obtained with each of the primer pairs for a given locus were combined and electrophoresed together in the same gel lane (Figure 6B shows the results for axenic strains). The identities of the bands were confirmed by Southern hybridization with a flanking region probe (middle panel, Figure 6B) or an EhSINE1 probe (bottom panel, Figure 6B). DNA from strains HM-1:IMSS and Rahman gave the expected amplicon with each primer pair, except for the 1.4 kb band with primers 13.1 F and 13.2 R expected from HM-1:IMSS, which could not be amplified efficiently. Hence HM-1:IMSS locus 13 was identified by the 0.2 kb 13.1 F/SINE R product. Results with the seven axenic strains showed that EhSINE1 was present at all three loci in strains MS84-1373 and MS27-5030. In this respect they behaved like HM-1:IMSS. However, primer set 17.2 F-17 .2 R could not amplify MS84 and primer set 17.2 R-SINE R could not amplify MS27, indicating that they were not identical to HM-1:IMSS at locus 17. Single nucleotide mutations in the flanking sequences could lead to sequence polymorphisms in these regions and give the observed result due to loss of primer recognition. Since the sequence of this region is not known in these other strains, an explanation for this result would have to await further sequence data. Similarly, strain HK-9 resembled Rahman at all three loci in terms of EhSINE1 occupancy but belonged to a third category since at locus 13 it repeatedly failed to give the expected amplicon size with primer pair 13.1 F-13.2R although the expected amplicon was obtained with primer pair 13.1 F-13.1R (Figure 7A). Strains PVBM08B and PVBM08F were like Rahman at locus 17 and like HM-1:IMSS at loci 13 and 19. Strain MS96-3382 was like Rahman at loci 13 and 17. However, genome sequence analysis (AmoebaDB) showed the presence of a 397 bp SINE sequence (truncated from both ends) at locus 17 in this strain. Since the PCR and Southern data for this locus were unambiguous we are inclined to believe that, as mentioned earlier (Figure 5), the discrepancy between our data and AmoebaDB may be due to sequence assembly problems. Strain 200:NIH was like Rahman at loci 17 and 19. Thus, based on the presence and absence of SINE1, and the amplicons obtained with each primer pair at these three loci, the axenic strains could be divided into five genotypes (Table 3).

**Table 3:** **Categorization of *E. histolytica *strains**

**Locus**	**13**	**17**	**19**	**Genotype**
**Strain**	**type**	**type**	**type**	
**PVBM08B**	H	R	H	HRH
**MS96 -3382**	R	R	H	RRH
**MS84-1373**	H	N	H	HNH
**MS27-5030**	H	N	H	HNH
**HK-9**	N	R	R	NRR
**PVBM08F**	H	R	H	HRH
**200:NIH**	H	R	R	HRR
25591	H	R	N	HRN
26825	N	H	N	NHN
27749	H	N	R	HNR
28577	H	H	H	HHH
30325	H	H	H	HHH
32083	N	N	R	NNR
32223	H	R	H	HRH
32257	H	H	H	HHH
33526	H	H	H	HHH
34153	H	R	R	HRR
34180	H	H	H	HHH
34276	H	R	R	HRR
581	N	N	H	NNH
654	N	N	H	NNH
812	H	N	R	HNR
878	N	R	R	NRR

Axenic strains (PVBM08B - 200:NIH; boldfaced) and xenic cultures (25591–878) of *E. histolytica* were categorized into HM-1:IMSS (H)/Rahman (R)/neither (N) type based on amplification patterns at loci 13, 17, and 19. Abbreviation used for axenic strains PVBM08B, MS96-3382, MS84-1373, MS27-5030, PVBM08F, 200:NIH in the figures as PVB, MS96, MS84, MS27, PVF and NIH respectively.

The same primer pairs were used for analysis of 16 clinical isolates of *E. histolytica* (Figure 7, Additional file 8: Table S3). The results are summarized in Table 3. The amplicons were clearly visible only after Southern hybridization for most clinical isolates. The results clearly show mosaic patterns in the three loci, displaying characters of both HM-1:IMSS and Rahman in many strains.

To sum up the above data, a total of 25 *E. histolytica* strains were used in this study, of which HM-1:IMSS contains EhSINE1 at all three loci (HHH), while Rahman lacks the element at all three loci (RRR). In the remaining 23 *E. histolytica* strains (including axenic and xenic clinical isolates), EhSINE1 was absent at loci 13, 17 and 19 in 7, 10 and 8 strains respectively. Based on the presence/absence of EhSINE1, and amplicons obtained with the primer pairs at these three loci, the 23 strains were categorized into eleven genotypes (Table 3). Based on SINE occupancy there can only be eight combinations at the three loci (i.e. 2^3^). Additional variations (designated N, which are neither H nor R) have come about due to alterations in flanking sequences leading to loss of primer recognition sites. In the 23 strains tested the most frequent combination was HHH (5 strains) followed by HRR and HRH (3 strains each) and HNH, NRR, HNR and NNH (2 strains each). The use of multiple loci for strain identification is preferred [23,25] as a single locus cannot differentiate all the strains. The results obtained by our method corroborated with the data from tRNA-STRs. Both methods distinguished the strains HM-1:IMSS, Rahman, 200:NIH and HK-9 from one another [20,25,26] and gave the same pattern for strains PVB and PVF (Clark C.G., unpublished observation). Thus our results suggest that in principle genomic distribution of SINEs can be used as a valid method for typing of *E. histolytica* strains.

Although SINEs are mobile genetic elements, their mobilization in present-day *E. histolytica* is probably a very infrequent event. This can be inferred from the fact that most genomic copies of the EhLINE1 retrotransposon (which provides the machinery for EhSINE1 mobilization through retrotransposition) are inactive. We have shown experimentally that the retrotransposition activity in these cells is very low or absent [39]. Therefore the genomic location of SINEs in a given strain is stable enough to be used as a strain-specific signature.

### Bioinformatic analysis of EhSINE2 copies

**Figure 8:**
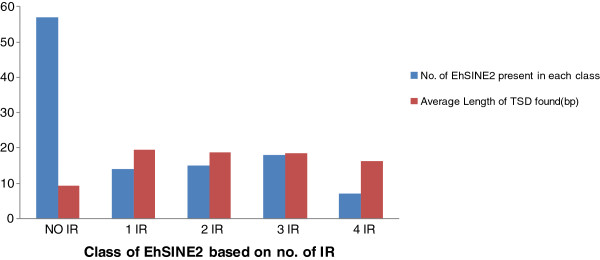
**Classification of EhSINE2.** 119 EhSINE2 copies were extracted from the *E. histolytica* HM-1:IMSS database (length > 400 bp and similarity >70% with the consensus EhSINE2) and analyzed for IR; 111 could be categorized according to number of internal repeats, represented by the bars in Blue. The rest were excluded due to having a single copy in the database or having only a fraction of an IR in the SINE2. Correlation of TSD length and number of IR. Out of 119 SINE2s analyzed, TSDs were found in 92 cases (77.31%). All 92 examples with a TSD were analyzed for the number of IRs and average TSD length was plotted against IR number, represented by the bars in Red.

Although a detailed bioinformatic analysis of EhSINE1 has been published [30], a similar analysis of EhSINE2 has not been reported. Therefore we decided to carry out an analysis of EhSINE2 using the approach that has been described for EhSINE1. All sequences that displayed similarity of more than 70% with the consensus sequence and a length of more than 400 bp were extracted from the genome sequence of *E. histolytica* available at NCBI (total 119). These were analysed for internal repeats (IR) by using Tandem repeat finder [40]. Some of the EhSINE2 sequences also contained IRs, as reported in EhSINE1 (which contains 26–27 bp IRs). EhSINE2 copies could be categorized into distinct classes based on number of IRs (Figure 8). The class with three IRs was the most common, followed by those with two, one and four IRs, respectively (Figure 8). A single copy each of 5 and 13 IR-containing EhSINE2s was also found. About half the EhSINE2 copies either lacked an IR or contained only a fragment of one. We also found one copy each of EhSINE2s that matched the length expected of copies with 1 IR and 3 IR, but in fact contained no IR at all. These observations are similar to EhSINE1 where it was reported that 60% of the copies had either no IR or had the appropriate length for 3 IR but only one out of three IRs was recognizable [30].We analyzed the IR sequences of all EhSINE2 copies and extracted 150 IR sequences; the majority were 20 bp in length except four, in which the IR was 13–14 bp. A common motif present in these IR sequences was identified by the online motif search tool, MEME to be AATGAATAACAATACACG/CTT/C.

As already mentioned, retrotransposition is accompanied by generation of TSDs. Newly retrotransposed copies are expected to be flanked by identical TSDs, while over time these accumulate mutations, become shorter in length and are finally unrecognizable. Therefore length of TSDs may be a marker of age of SINEs [29]. We analyzed the TSDs of all 119 EhSINE2 copies, and could find TSD in 97 cases. The longest TSDs (ranging in sizes from 16–20 bp) were found in elements with IRs, while copies lacking intact IRs displayed smaller TSDs, in the range of 8–9 bp (Figure 8). This suggests that copies lacking IR may be older and may have suffered loss of IR sequences subsequent to retrotransposition. In the case of EhSINE1, the 2 IR-containing copies were reported to be the most recently transposed elements as they had longer TSDs than the other copies [30]. The TSDs of 81 EhSINE2 sites (excluding those below 8 bp in length) were analyzed by MEME. All 81 TSDs showed the consensus motif T(T/C)T(T/C)TN(A/T)T, suggesting a high percentage of pyrimidines is needed at the insertion point.

## Conclusion

SINE elements are useful genomic markers due to their wide occurrence and property of irreversible re-integration in the host genome [15]. The loss of SINEs from genomic loci is a rare event and is generally accompanied by changes in flanking sequences as well [41]. Therefore, as stated earlier, SINEs are better suited to establish genealogies below the species level with minimal assumptions compared with other standard markers, such as microsatellites, RFLPs, and SNPs, which can result from independent mutations at different times that are not inherited from a common ancestor [16,42-46]. For this reason the analysis of SINE occupancy in *E. histolytica* strains reported here will be significant to establish intraspecific relationships.

Retrotransposons are known to influence the expression of genes in their vicinity by various mechanisms, including silencing by heterochromatinization, up-regulation by providing alternate promoters, and novel expression patterns through alternative splicing and polyadenylation [47-50]. Thus the gain or loss of EhSINE1 element from a genomic locus could potentially influence the phenotype of the organism in a profound manner. For this reason the strain typing method used here has a potential to reveal loci that may be associated with different phenotypes, including the virulence properties of the parasite. However more samples need to be tested to provide a correlation between virulence and genotype. A combination of rapid genome sequencing and expression analysis from a variety of clinical isolates of *E. histolytica* by NGS will reveal whether retrotransposons in *E. histolytica* have the ability to influence neighboring gene expression. This method of strain typing based on retrotransposon occupancy could then have physiological relevance.

## Competing interests

The authors declare that they have no competing interests.

## Authors’ contributions

Conceived and designed the experiments: SB and VK. Performed the experiments: VK, VB, SP Analyzed the data: SB AB VK. Computational work done: RR, VK, VB, SP. Contributed reagents/materials/analysis tools: SB, LRI, JP, JJV, CGC. Wrote the paper: SB AB VK. Principal investigator: SB. Reviewed and commented the paper: CGC AB JP LRI JJV. All authors read and approved the final manuscript.

## Supplementary Material

Additional file 1: Figure S1 Description: Schematic representation of flanking genes, EhSINE1/EhSINE2, and position of primers on the *E. histolytica* HM-1:IMSS scaffolds containing loci 13, 17, 19, 42, 18 and 50. The thin line represents the scaffold, arrowheads denote the different primers, solid boxes represent genes, hollow boxes represent a EhSINE (arrow indicates orientation) and the grey box denotes any repetitive element other than a SINE. Numbers on vertical lines indicate the position of genes and EhSINE on the scaffold. Click here for file

Additional file 2: Table S1 Description: Expected amplicon size with each primer pair from genome assemblies. Click here for file

Additional file 3: Figure S2 Description: Analysis of locus 42: Locus 42 was amplified from the genomic DNA of *E. histolytica* HM-1:IMSS and Rahman with the locus-specific primers followed by Southern blotting and hybridization with a locus 42-specific probe (3.7 kb amplicon from the genomic DNA of HM-1:IMSS). Click here for file

Additional file 4: Table S2 Description: Detailed analysis of loci 13, 17, 19, 42, 18 and 50 in sequenced strains (AmoebaDB). Click here for file

Additional file 5: Figure S4 Description: Schematic representation of locus 17 HM-1:IMSS and Rahman (AmoebaDB): Intact, dotted, broken line, hollow boxes and arrowheads represent similar features to those described in Additional file 7: Figure S3. Scaffold DS571247 contains locus 17 of HM-1:IMSS. The corresponding locus in Rahman is present in EhRm_scaffold00561. The EhSINE1 region, including 300 bp upstream sequence, in HM-1:IMSS is undefined in Rahman (represented by a thin dotted line). A stretch of 84 bp of EhSINE1 from the 5′ end was retained in Rahman (represented by small hollow box). As mentioned in the text and figure 5 assembly of Rahman sequence at the SINE region is erroneous in the database. In fact the entire EhSINE1 sequence is missing in Rahman. Click here for file

Additional file 6: Figure S5 Description: Schematic representation of locus 19 HM-1:IMSS and Rahman (AmoebaDB): Intact, dotted and broken lines, hollow boxes and arrowheads represent similar features to those described in Additional file 7: Figure S3. Scaffold DS571226 contains locus 19 of HM-1:IMSS. The corresponding Rahman locus is present in one major scaffold (EhRm_scaffold00536) and three small unassembled contigs (EhRm_contig00303, EhRm_contig00523, EhRm_contig21711), which are represented by red, purple and blue lines and a green box respectively. Ehrm_scaffold00536 has a large undefined region (Ns) where these small contigs are located. Click here for file

Additional file 7: Figure S3 Description: Schematic representation of locus 42 in HM-1:IMSS and Rahman (AmoebaDB): Intact lines represent regions that show homology in the two strains (some mismatches have been ignored). The dotted line represents the missing EhSINE1 sequence in Rahman and the hollow box represents EhSINE1 in HM-1:IMSS. The black line represents the Scaffold containing locus 42 of HM-1:IMSS. Red and purple lines and the green box represent EhRm_scaffold00892, EhRm_scaffold00027, EhRm_contig21200, respectively, which contain the corresponding locus in Rahman. Boxes represent the upstream hypothetical protein and downstream mannosyltransferase protein genes. Arrowheads represent the primers and G represent the last nucleotide of the primer (the position of which is indicated in the HM-1:IMSS scaffold) while C represent the mismatched nucleotide at the respective position in Rahman. The blue arrowhead shows the proposed position of the primer in the Rahman scaffold where it may anneal to give the observed amplicon (~5.2 kb) (ACG (blue) represents the last 3 nucleotides of 42.1 F matching this position in the Rahman scaffold). Downstream of EhSINE1 there is a truncated 1.2 kb EhLINE1 sequence which is partly present in two scaffolds of Rahman. Numbers above and below the lines represent the respective positions in the scaffolds/contigs of HM-1:IMSS and Rahman, as well as identifying the position of EhSINE1, genes and the other repetitive region in the loci in the two genomes. Broken lines at the end of scaffold indicate the further extension of scaffolds beyond the region depicted. Click here for file

Additional file 8: Table S3 Description: List of xenic isolates. Click here for file
