# Supplementary figures and images for: Genomic distribution of SINEs in Entamoeba histolytica strains: implication for genotyping
Source: BMC Genomics. 2013 Jul 1;14:432. doi: 10.1186/1471-2164-14-432 (PMC3716655; doi:10.1186/1471-2164-14-432)

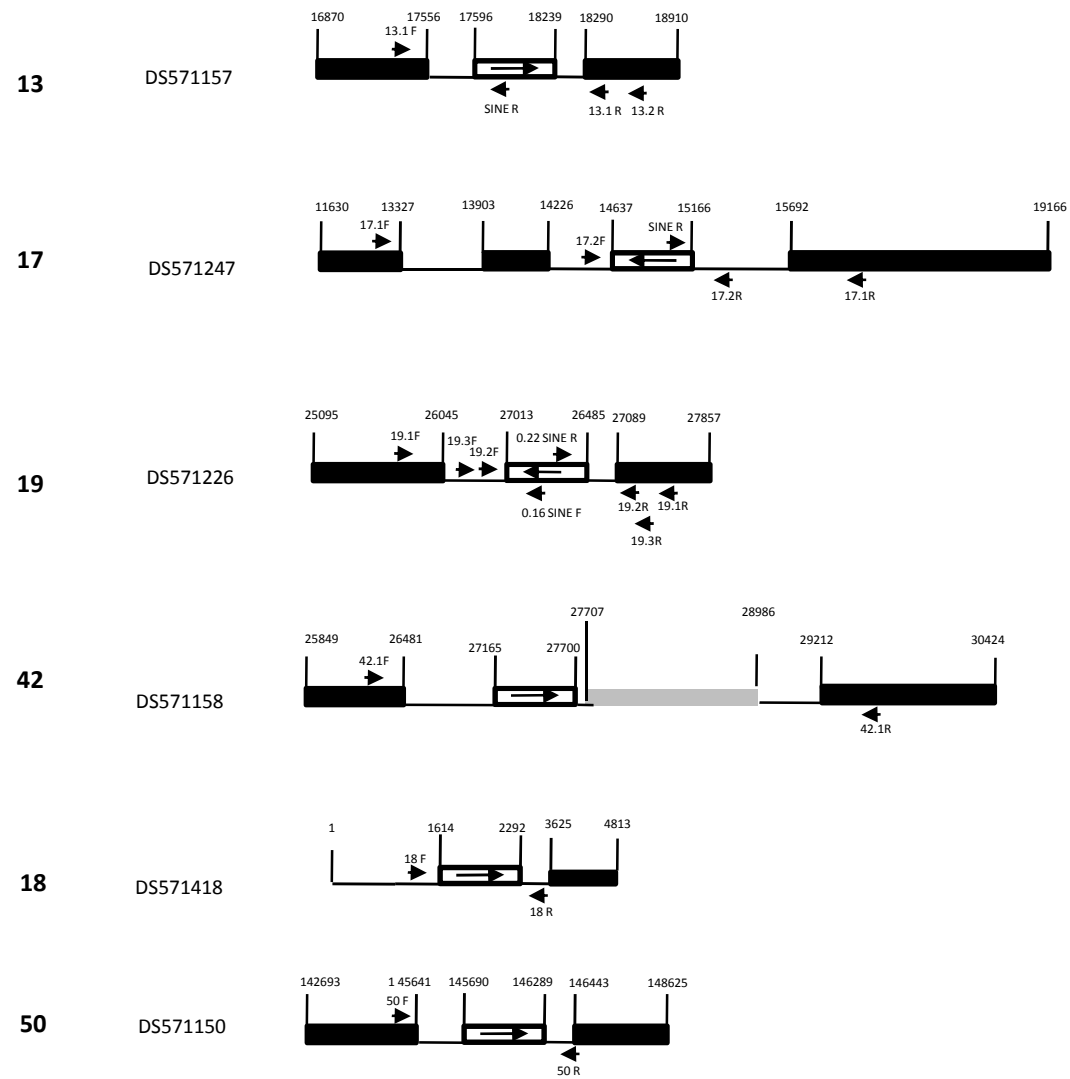

Figure S1

Supplement: Additional file 1: Figure S1 — Description: Schematic representation of flanking genes, EhSINE1/EhSINE2, and position of primers on the E. histolytica HM-1:IMSS scaffolds containing loci 13, 17, 19, 42, 18 and 50. The thin line represents the scaffold, arrowheads denote the different primers, solid boxes represent genes, hollow boxes represent a EhSINE (arrow indicates orientation) and the grey box denotes any repetitive element other than a SINE. Numbers on vertical lines indicate the position of genes and EhSINE on the scaffold. [file 1471-2164-14-432-S1.pdf]

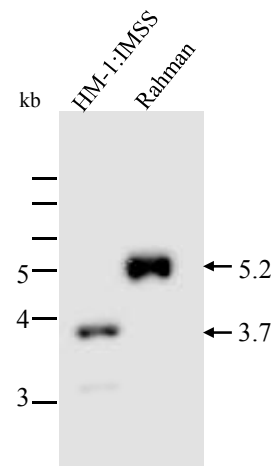

Figure S2

Supplement: Additional file 3: Figure S2 — Description: Analysis of locus 42: Locus 42 was amplified from the genomic DNA of E. histolytica HM-1:IMSS and Rahman with the locus-specific primers followed by Southern blotting and hybridization with a locus 42-specific probe (3.7 kb amplicon from the genomic DNA of HM-1:IMSS). [file 1471-2164-14-432-S3.pdf]

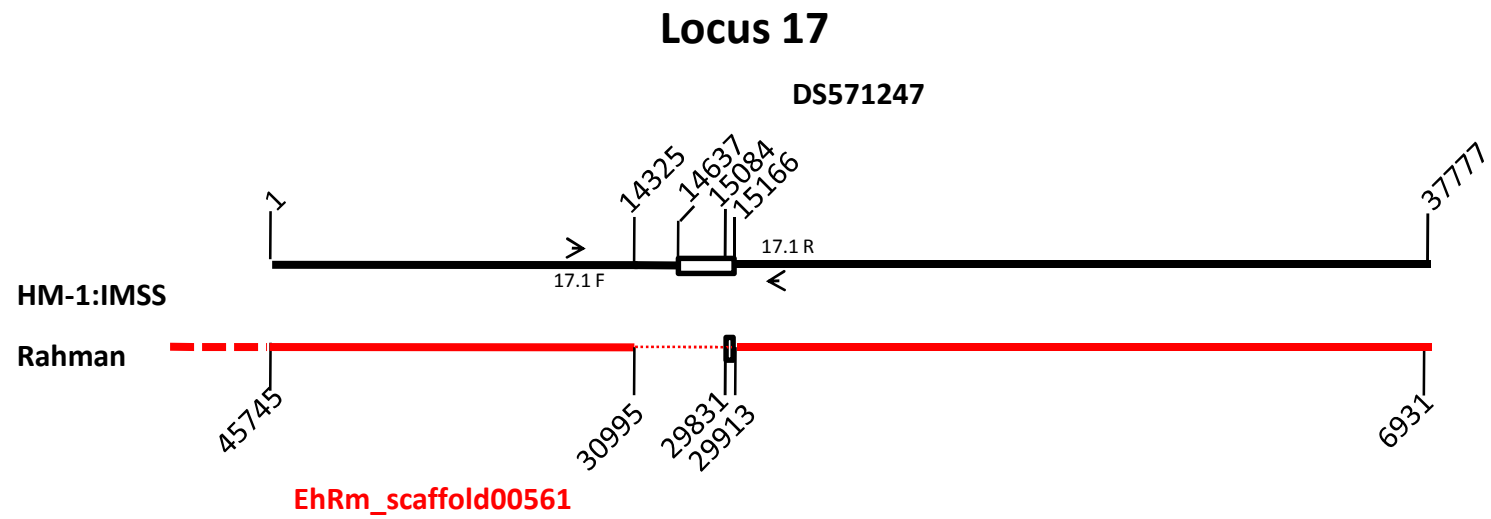

Figure S4

Supplement: Additional file 5: Figure S4 — Description: Schematic representation of locus 17 HM-1:IMSS and Rahman (AmoebaDB): Intact, dotted, broken line, hollow boxes and arrowheads represent similar features to those described in Additional file 7: Figure S3. Scaffold DS571247 contains locus 17 of HM-1:IMSS. The corresponding locus in Rahman is present in EhRm_scaffold00561. The EhSINE1 region, including 300 bp upstream sequence, in HM-1:IMSS is undefined in Rahman (represented by a thin dotted line). A stretch of 84 bp of EhSINE1 from the 5′ end was retained in Rahman (represented by small hollow box). As mentioned in the text and figure 5 assembly of Rahman sequence at the SINE region is erroneous in the database. In fact the entire EhSINE1 sequence is missing in Rahman. [file 1471-2164-14-432-S5.pdf]

## Locus 19

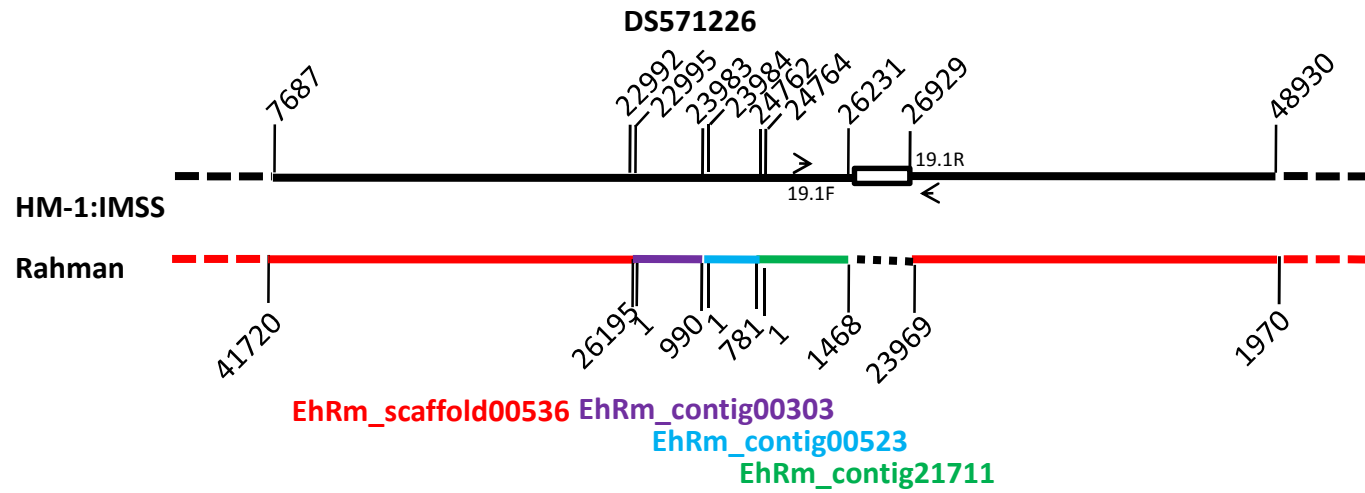

Figure S5

Supplement: Additional file 6: Figure S5 — Description: Schematic representation of locus 19 HM-1:IMSS and Rahman (AmoebaDB): Intact, dotted and broken lines, hollow boxes and arrowheads represent similar features to those described in Additional file 7: Figure S3. Scaffold DS571226 contains locus 19 of HM-1:IMSS. The corresponding Rahman locus is present in one major scaffold (EhRm_scaffold00536) and three small unassembled contigs (EhRm_contig00303, EhRm_contig00523, EhRm_contig21711), which are represented by red, purple and blue lines and a green box respectively. Ehrm_scaffold00536 has a large undefined region (Ns) where these small contigs are located. [file 1471-2164-14-432-S6.pdf]

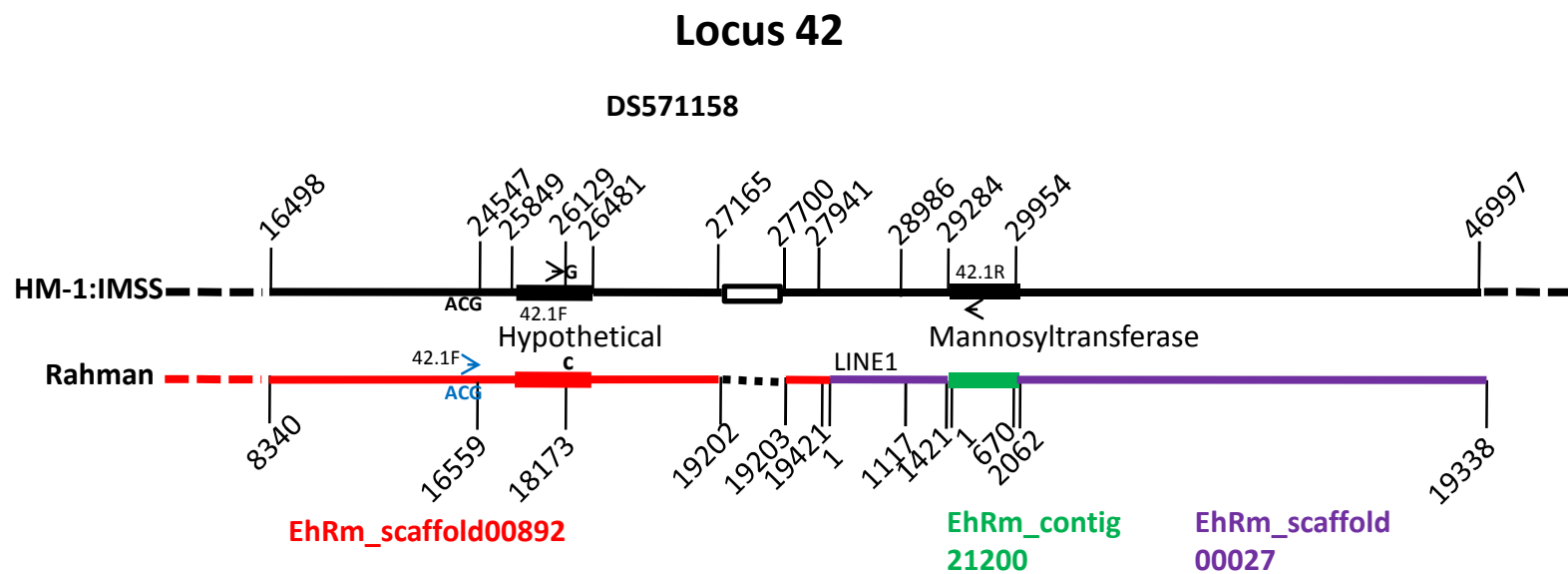

Figure S3

Supplement: Additional file 7: Figure S3 — Description: Schematic representation of locus 42 in HM-1:IMSS and Rahman (AmoebaDB): Intact lines represent regions that show homology in the two strains (some mismatches have been ignored). The dotted line represents the missing EhSINE1 sequence in Rahman and the hollow box represents EhSINE1 in HM-1:IMSS. The black line represents the Scaffold containing locus 42 of HM-1:IMSS. Red and purple lines and the green box represent EhRm_scaffold00892, EhRm_scaffold00027, EhRm_contig21200, respectively, which contain the corresponding locus in Rahman. Boxes represent the upstream hypothetical protein and downstream mannosyltransferase protein genes. Arrowheads represent the primers and G represent the last nucleotide of the primer (the position of which is indicated in the HM-1:IMSS scaffold) while C represent the mismatched nucleotide at the respective position in Rahman. The blue arrowhead shows the proposed position of the primer in the Rahman scaffold where it may anneal to give the observed amplicon (~5.2 kb) (ACG (blue) represents the last 3 nucleotides of 42.1 F matching this position in the Rahman scaffold). Downstream of EhSINE1 there is a truncated 1.2 kb EhLINE1 sequence which is partly present in two scaffolds of Rahman. Numbers above and below the lines represent the respective positions in the scaffolds/contigs of HM-1:IMSS and Rahman, as well as identifying the position of EhSINE1, genes and the other repetitive region in the loci in the two genomes. Broken lines at the end of scaffold indicate the further extension of scaffolds beyond the region depicted. [file 1471-2164-14-432-S7.pdf]
