# Supplementary material for: Genomic distribution of SINEs in Entamoeba histolytica strains: implication for genotyping
Source: BMC Genomics. 2013 Jul 1;14:432. doi: 10.1186/1471-2164-14-432 (PMC3716655; doi:10.1186/1471-2164-14-432)
Supplement: Additional file 2: Table S1 — Description: Expected amplicon size with each primer pair from genome assemblies. [file 1471-2164-14-432-S2.pdf]

Table S1. Expected amplicon size with each primer pair from genome assemblies

Locus	Primers	Expected size (kb)	
		HM-1:IMSS	Rahman
13	**13.1 F/13.2 R**	1.4	0.83
	**13.1 F/** SINE R	0.21	-
	**13.1 F/**13.1R	1.1	0.42
17	17.1 F/17.1 R	2.53	2.0
	**17.2 F/17.2 R**	0.79	0.23
	**17.2 R/** SINE R	0.13	-
19	19.1 F/ 19.1 R	2.1	1.55
	**19.3 F/**19.3 R	0.91	0.32
	**19.3 F/**0.16 SINE F	0.66	-
42	42.1 F/ 42.1 R	3.7	5.2
18	18 F/18 R	0.96	0.24
50	50 F/50 R	0.86	0.16

Primers not matching *E. dispar* are in boldface.
