# Supplementary material for: Genomic distribution of SINEs in Entamoeba histolytica strains: implication for genotyping
Source: BMC Genomics. 2013 Jul 1;14:432. doi: 10.1186/1471-2164-14-432 (PMC3716655; doi:10.1186/1471-2164-14-432)
Supplement: Additional file 4: Table S2 — Description: Detailed analysis of loci 13, 17, 19, 42, 18 and 50 in sequenced strains (AmoebaDB). [file 1471-2164-14-432-S4.pdf]

Table S2. Detailed analysis of loci 13, 17, 19 42, 18 and 50 in sequenced strains (AmoebaDB).

Eh strain	locus	ID	SINE occupancy	start	End	Remarks
DS4	13	EHDS4_4213	+	84933	85542	
	17	EHDS4_2840	+	84936	85541	
	19	EHDS4_3346	+	26367	26920	
	42	EHDS4_3522	+	21707	21978	Truncated (271 bp of EhSINE1)
	SINE2					
	18	EHDS4_4206	+	1601	2278	
	50	EHDS4_3252	+	145133	145728	Eh SINE2 slightly truncated from both sides
HM1:CA	13	EHHM1_CA_4213	+	79360	79884	100 bp deletion within EhSINE1
	17	EHHM1_CA_2840	+	13367	13921	
	19	EHHM1_CA_3346	+	22600	23100	
	42	EHHM1_CA_3522	+	25911	26448	30 bp deletion within EhSINE1at 3' end
	SINE2					
	18	EHHM1_CA_4206	+	1570	2251	
	50	EHHM1_CA_3252	+	136204	136749	Deletion from within EhSINE2 (414-532)
KU27	13	EHKU27_4213	+	84501	85105	
	17	EHKU27_2840	+	14648	14878	Truncated from 5' end (230 bp of EhSINE1)
	19	EHKU27_3346	+	26048	26601	
	42	EHKU27_3522	+	20600	21115	EhSINE1 slightly truncated from 5' end
	SINE2					
	18	EHKU27_4206	+	1588	2240	Small (20bp) deletion within EhSINE2 (3 IR instead of 4 IR)
	50	EHKU27_3252	+	145081	145650	Truncation from 5' end and 13 bp deletion within EhSINE2
KU48	13	EHKU48_4213	+	72608	73198	40 bp deletion within EhSINE1
	17	EHKU48_2840	+	13293	13847	
	19	EHKU48_3346	+	17941	18501	
	42	EHKU48_3522	+	21600	22120	
	SINE2					
	18	EHKU48_4206	-	NA	NA	
	50	EHKU48_3252	+	125979	126087	Only 109 bp of EhSINE2 present
KU50	13	EHKU50_4213	+	47531	48136	Slightly truncated from 5' end and 10 bp deletion within EhSINE1
	17	EHKU50_2840	+	4860	5439	Truncation from 3' end of EhSINE1
	19	EHKU50_3346	-	7929	7885	Only 40 bp of EhSINE1present
	42	EHKU50_3522	+	11229	11469	EhSINE1truncated from both side (240 bp of EhSINE1 present)
	SINE2					
	18	EHKU50_4206	-	NA	NA	
	50	EHKU50_3252	+	145787	146189	Truncated from 5' end of EhSINE1
MS96	13	EHMS96_4213	-	82910	82990	Only 80 bp of EhSINE1present
	17	EHMS96_2840	+	14705	14308	Truncated from both side (397 bp of EhSINE1 present)
	19	EHMS96_3346	+	26383	26937	
	42	EHMS96_3522	+	24410	24950	EhSINE1 slightly truncated at 5' end
	SINE2					
	18	EHMS96_4206	+	1615	2236	Slight (37 bp) deletion within EhSINE2
	50	EHMS96_3252	-	NA	NA	
Rahman	13	EhRm_Scaffold01127	-	NA	NA	
	17	EhRm_scaffold00561	-	29831	29913	Only 90 bp EhSINE1 present
	19	EhRm_scaffold00536<br>EhRm_contig21711	-	NA	NA	
	42	EhRm_scaffold00892	-	NA	NA	
	SINE2					
	18	EhRm_scaffold00754	-	NA	NA	
	50	EhRm_scaffold00159	-	NA	NA	

Scaffolds/Contigs shaded in yellow represent poor assembly (having gaps). SINE elements of <100 bp are considered to be negative (-) occupancy. NA- not applied. As mentioned in the text and figure 5 assembly of Rahman sequence at the SINE region is erroneous in the database. In fact the entire EhSINE1 sequence is missing in Rahman.
