# Supplementary material for: Genomic distribution of SINEs in Entamoeba histolytica strains: implication for genotyping
Source: BMC Genomics. 2013 Jul 1;14:432. doi: 10.1186/1471-2164-14-432 (PMC3716655; doi:10.1186/1471-2164-14-432)
Supplement: Additional file 8: Table S3 — Description: List of xenic isolates. [file 1471-2164-14-432-S8.pdf]

Table S3. List of xenic isolates

Lab Number	Sex	Diagnosis	Country	Continent
25591	M	liver abscess	unknown	South America
26825	F	diarrhea	Rwanda/Congo	Africa
27749	F	unknown	Tibet/Nepal	Asia
28577	F	dysentery	Guinea	Africa
30325	M	diarrhea	Indonesia	Asia
32083	M	liver abscess	Nigeria	Africa
32223	M	liver abscess	Indonesia	Asia
32257	F	no symptoms	Bangladesh	Asia
33526	M	liver abscess	El Salvador	South America
34153	unknown	unknown	unknown	unknown
34180	F	unknown	unknown	unknown
34276	M	unknown	unknown	unknown
581	F	unknown	India	Asia
654	M	unknown	India	Asia
812	F	unknown	India	Asia
878	M	unknown	India	Asia

Likely geographic source and the clinical diagnosis of the patient from which the *E. histolytica* were isolated.
